# Dosimetry Analysis of ^177^Lu-PSMA-I&T in Patients with Low-Volume Oligometastatic Hormone-Sensitive Prostate Cancer: A Secondary Analysis of the LUNAR Trial

**DOI:** 10.2967/jnumed.125.271467

**Published:** 2026-07

**Authors:** Zachary Ells, Catherine Meyer, Koichiro Kimura, Holly Wilhalme, Minsong Cao, Vinicius B. Ludwig, Lena M. Unterrainer, David Sennung, Rejah Nabong, Carol Felix, Luca F. Valle, Anthony Daley, Johannes Czernin, Magnus Dahlbom, Amar U. Kishan, Jeremie Calais

**Affiliations:** 1 Department of Nuclear Medicine and Theranostics, Ahmanson Translational Theranostics Division, David Geffen School of Medicine, UCLA, Los Angeles, California;; 2 Department of Medicine Statistical Core, UCLA, Los Angeles, California;; 3 Department of Radiation Oncology, UCLA, Los Angeles, California; and; 4 Department of Nuclear Medicine, TUM Klinikum, Rechts der Isar, Technical University of Munich, School of Medicine and Health, Munich, Germany

**Keywords:** ^177^Lu, PSMA, dosimetry, theranostics, SBRT, prostate cancer, ^177^Lu-PSMA-I&T, oligorecurrent

## Abstract

The phase 2 LUNAR trial randomized (1:1) patients with oligorecurrent hormone-sensitive prostate cancer to neoadjuvant [^177^Lu]Lu-PSMA-I&T (2 cycles, 6.8 GBq) followed by stereotactic body radiotherapy (SBRT) versus SBRT alone. [^177^Lu]Lu-PSMA-I&T before SBRT was well tolerated and significantly improved PSMA PET/CT–based progression-free survival compared with SBRT alone. Here, we report the estimated absorbed doses (AD) of [^177^Lu]Lu-PSMA-I&T to organs at risk and lesions. **Methods:** This analysis was conducted on all 45 patients randomized to the investigational arm. Quantitative SPECT/CT images were acquired at 4, 24, and 72–96 h postinjection of cycle 1. Kidneys, salivary and lacrimal glands, and liver were delineated with deep learning–assisted segmentation, whereas lumbar vertebrae were manually segmented as a surrogate for bone marrow. Planned target volumes were transferred from the SBRT plans to the SPECT/CT series. Registration between time points was manually verified for each segmentation. ADs were estimated using a multiple-time-point voxel-based schema. Time–activity data were fit using a monoexponential function. Partial-volume effects were corrected using volume-specific phantom-based recovery coefficients. **Results:** In the 45 patients included, the median prostate-specific antigen was 1.10 ng/mL (range, 0.16–14.70 ng/mL). In total, 123 lesions total were identified, with a median per patient of 2 (range, 1–9). Median whole-body total tumor volume was 14.5 cm^3^ (range, 1.9–145.9 cm^3^). Median SUV_max_ on baseline PSMA PET/CT and 24-h SPECT/CT was 3.49 (range, 0.59–45.30) and 0.72 (range, 0.02–34.24), respectively. The AD to the kidneys, parotids, submandibulars, lacrimals, liver, and bone marrow were 0.35 ± 0.10, 0.20 ± 0.10, 0.24 ± 0.10, 0.70 ± 0.49, 0.03 ± 0.01, and 0.005 ± 0.002 Gy/GBq, respectively. The mean dose to bone (*n* = 38), lymph node (*n* = 82), and soft tissue (*n* = 3) lesions were 0.19 ± 0.42, 0.46 ± 0.81, and 0.30 ± 0.38 Gy/GBq, respectively. **Conclusion:** The ADs from [^177^Lu]Lu-PSMA-I&T to organs at risk were consistent with prior reports, supporting the safety in patients with oligorecurrent hormone-sensitive prostate cancer. There was substantial heterogeneity in lesion AD estimates on both inter- and intrapatient levels. Because of the limited spatial resolution of SPECT, partial-volume effects can underestimate the AD in small volumes. Nevertheless, 2 neoadjuvant cycles of [^177^Lu]Lu-PSMA-I&T before SBRT prolonged progression-free survival, consistent with effective treatment of occult disease beyond imaging detectability.

## Visual Abstract

**Figure:**
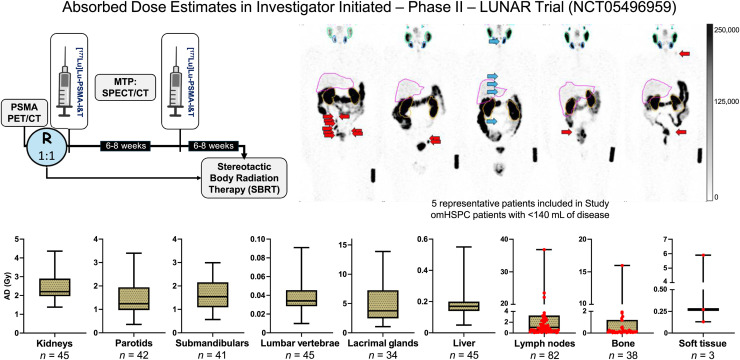

Prostate-specific membrane antigen (PSMA)–targeted radiopharmaceutical therapy (RPT) labeled with ^177^Lu is approved in the United States to treat patients with metastatic castration-resistant prostate cancer both in the postchemotherapy and prechemotherapy settings based on the VISION and PSMAfore trials, respectively (1,2). The favorable physical characteristics of ^177^Lu for clinical applications can be briefly summarized as the following: β^−^ emissions (average travel path, 0.670 mm in soft tissue), a half-life of 6.7 d, and imageable primary γ-emissions at 113 keV (6.6%) and 208 keV (11%) (3).

Metastasis-directed therapy (e.g., stereotactic body radiation therapy [SBRT]) has been shown to prolong progression-free survival (PFS) compared with observation in patients with oligometastatic hormone-sensitive prostate cancer (omHSPC) (4–7). The addition of [^177^Lu]Lu-PSMA RPT to SBRT in patients with omHSPC may treat not only the detectable disease but also microscopic disease (8). Toxicity and quality of life outcomes in these patients with earlier disease stage were found to be similar across trials (prospective and retrospective) and heavily pretreated patient populations (9–13). The phase 2 LUNAR trial (^177^Lu-PSMA neoadjuvant to stereotactic ablative radiotherapy) randomized (1:1) patients with omHSPC with 1–5 tumor targets visible by PSMA PET to receive neoadjuvant [^177^Lu]Lu-PSMA-I&T (2 cycles) before SBRT versus SBRT alone. Clinical outcomes were reported previously (14). Briefly, [^177^Lu]Lu-PSMA-I&T plus SBRT significantly improved PSMA PET/CT–based PFS compared with SBRT alone (median, 17.6 vs. 7.4 mo; hazard ratio, 0.37) and had comparable adverse events between groups. As patients are treated at earlier disease stages with RPT, adverse events are a potential concern; the most common events associated with [^177^Lu]Lu-PSMA RPT include fatigue, mild xerostomia, and nausea (2). Other concerns include nephrotoxicity and bone marrow toxicity.

This secondary analysis of the LUNAR trial evaluates [^177^Lu]Lu-PSMA-I&T dosimetry in omHSPC, reporting absorbed dose (AD) estimates to both organs at risk (OAR) and lesions.

## MATERIALS AND METHODS

The LUNAR trial (NCT05496959) was a single-center, investigator-initiated (sponsor UCLA, principal investigator Amar U. Kishan), randomized phase 2 study conducted at UCLA supported by POINT BioPharma and Lantheus Medial Imaging. The clinical trial protocol can be found in previous publications (14,15), and the detailed study design can be found in Supplemental Figure 1 (supplemental materials are available at http://jnm.snmjournals.org). The study was approved by the institutional review board of UCLA (22-000750), and all patients provided written informed consent.

In total, 45 patients with omHSPC with 1–5 targets visible by PSMA PET were randomized to the intervention arm, receiving 2 cycles of neoadjuvant [^177^Lu]Lu-PSMA-I&T at a 6–8-wk time interval, followed by SBRT 6–8 wk later, versus 42 patients receiving SBRT alone. Administered activity of [^177^Lu]Lu-PSMA-I&T at both cycles was 6.8 ± 10 % GBq (∼184 mCi). The PSMA PET/CT scans were reviewed by investigators to confirm eligibility. A post–[^177^Lu]Lu-PSMA-I&T PSMA PET/CT scan was acquired before SBRT. Enrollment criteria for LUNAR are summarized in Supplemental Table 1.

### SPECT Acquisition and Reconstruction Protocol

[^177^Lu]Lu-PSMA SPECT/CT scans were acquired at multiple time points (4-, 24-, and 72–96-h postinjection) after the first cycle only. Images were acquired on a Siemens Intevo camera using 3 bed positions from vertex to midthigh (60 projections/detector at 10 s/view, 208 ± 10 keV energy window with triple-energy window scatter correction, 256 × 256 matrix, and voxel size of 4.8 mm^3^), followed by a low-dose CT scan (130 kV, CareDose, 3-mm slice thickness). SPECT reconstruction was completed using MIM SPECTRA Quant (MIM Software Inc.) with 48 iterations, 1 subset, and no post filter applied (calibration factor of 9.98 cps/MBq) (16).

### Segmentation

The following after OARs were semiautomatically segmented using CT-based deep learning–assisted segmentation (Contour Protégé AI; MIM Software Inc.) from the 24-h postinjection scan: submandibular glands, parotids, lacrimal glands, liver, and kidneys. The bone marrow segmentation was completed manually using whole lumbar vertebrae (L1–L5) without cortical bone (−160–180 Hounsfield unit restriction).

For tumor segmentation, the planned target volumes (PTVs) defined during the SBRT treatment were used. Those segmentations were transferred to the 24-h SPECT/CT using CT–CT rigid-based registration. Tumor lesion segmentation was then visually verified by a nuclear medicine physician to ensure proper localization, with manual adjustment of the contours when necessary.

### SUV

All PET/CT images were reconstructed using non–time of flight, 2 iterations and 21 subsets, and model-based scatter correction. SUV_max_ was determined for each lesion on the baseline PET/CT, the 24-h postinjection SPECT/CT, and the post–[^177^Lu]Lu-PSMA-I&T PET/CT.

### SPECT Partial-Volume Correction

The National Electrical Manufacturers Association/International Electrochemical Commission body phantom was used to calculate recovery coefficients for fillable spheric inserts with diameters of 10, 13, 17, 22, 28, and 37 mm. The phantom was filled with a ^177^Lu sphere–to–background activity concentration ratio of 16:1 (2056:130 kBq/mL). SPECT acquisition and reconstruction parameters were identical to clinical routine as described. Volume-specific recovery coefficient factors for the salivary glands, lacrimal glands, and each lesion were individually determined using the generated activity recovery curve (17,18).

### AD Estimate

A multiple-time-point SPECT/CT dosimetry protocol using a voxel *S* value dose calculation method was used to calculate the AD to OAR and lesions (MIM Sureplan MRT version 7.3.5).

Manual verification of the individual segmentations coregistered between SPECT/CT time points was completed. All time–activity data were fit using a monoexponential function. A partial-volume correction was applied to the salivary glands, lacrimal glands, and lesions based on volume as described previously.

### Statistics

All reported ADs are in gray and refer to the calculation from cycle 1. Analysis between lesion volume and calculated AD estimates was evaluated using a 2-tailed nonparametric Spearman correlation. To evaluate hematologic toxicities, a composite measure was created to identify patients who experienced at least grade 1 toxicity in any of the following categories: anemia, lymphopenia, neutropenia, or thrombocytopenia.

The Wilcoxon rank-sum test was used to assess the distribution between toxicities and mean AD. The relationship between percent prostate-specific antigen change from baseline (dichotomized as <50% vs. ≥50%) and mean AD was also assessed using the Wilcoxon rank-sum test. All statistical analyses were performed using SAS version 9.4 (SAS Institute). Two-sided *P* values less than 0.05 were considered statistically significant.

## RESULTS

All 45 patients randomized to the interventional arm received [^177^Lu]Lu-PSMA-I&T and were included in this analysis. The average injected activity at cycle 1 was 7020 ± 208 MBq. Patient characteristics are defined in Table 1.

**TABLE 1.** Patient Characteristics (*n* = 45)

Parameter	Value
Age (y)	
Mean ± SD	71 ± 7
Median and range	70 (50–83)
Weight (kg)	
Mean ± SD	85 ± 14
Median and range	82 (58–124)
PSA (ng/mL)	
Mean ± SD	2.34 ± 3.38
Median and range	1.10 (0.16–14.70)
Prior therapy	
Androgen deprivation therapy	25 (56)
Androgen receptor signaling inhibitor	8 (18)
Chemotherapy	3 (7)
Metastasis-directed therapy	13 (29)
Number of lesions	
1	14 (31)
2–3	19 (42)
4–5	12 (27)
Staging	
Conventional imaging	
N0M0	34 (76)
N1M0	1 (2)
N0M1a	1 (2)
N0M1b	9 (20)
PSMA	
N1a/M1a	28 (62)
M1b	17 (38)

Qualitative data are number with percentage in parentheses.

**FIGURE 1.**
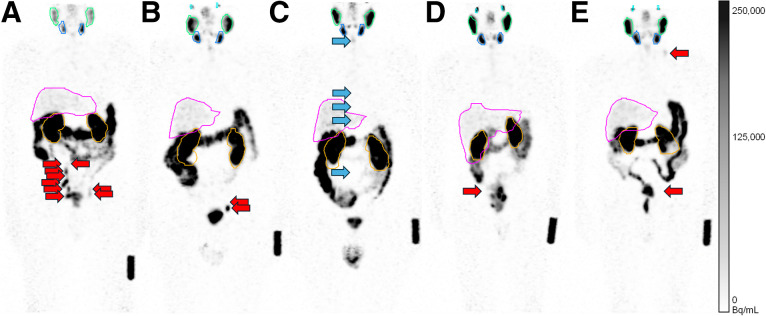
Five representative patient SPECT maximum-intensity projections at 24 h (anterior to posterior) included in interventional arm. All lesions shown with red arrows are LN-based, and lesions (only in (C)) highlighted with blue arrows are bone-based. (A) Patient with most individual lesions treated (*n* = 9). (B) Lesion with greatest tumor-to-background ratio included. (C) Greatest overall tumor volume (gross tumor volume–based, 36.0 mL; PTV-based, 145.9 mL). (D) Lowest overall tumor volume (gross tumor volume–based, 0.5 mL; PTV-based, 1.9 mL). (E) Distant disease.

All patients underwent multiple-time-point SPECT/CT imaging at a mean of 4.3, 23.9, and 90.0 h after [^177^Lu]Lu-PSMA-I&T injection. Twenty-eight patients (62%) had their final SPECT/CT acquisition at 72 h, whereas 17 (38%) patients had their final SPECT/CT acquisition at 96 h. Imaging time points are further described in Supplemental Table 2. SPECT/CT maximum-intensity projections of 5 representative patients at 24 h after [^177^Lu]Lu-PSMA-I&T injection are shown in Figure 1.

Patients included in this study were to have up to 5 target volumes that could be treated by SBRT. Four patients had multiple contiguous individual tumors within a single target that were judged clinically to represent one macroscopic volume but represented distinct tumors in this analysis. In these cases, the total number of lesions in the patients were 6, 6, 7, and 9.

### SPECT Partial-Volume Correction

The recovery curve model based on National Electrical Manufacturers Association/International Electrochemical Commission body phantom imaging is given by $f_{\mathrm{RC}}\left(V[\mathrm{mL}]\right)=1-{\left(1+{\left(\frac{V}{11.13}\right)}^{0.83}\right)}^{-1}$ (shown in Supplemental Fig. 2).

### SUV Characteristics

Baseline PET/CT was acquired for all 45 patients at a median of 58 d (interquartile range, 50–76 d) before [^177^Lu]Lu-PSMA-I&T injection. The second PET/CT was acquired at a median of 42 d (interquartile range, 36–50 d) after the second cycle of [^177^Lu]Lu-PSMA-I&T. The SUV_max_ was calculated for all 123 lesions at both PET/CT time points, and the 24-h postinjection SPECT/CT is shown in Supplemental Table 4.

On the 24-h postinjection SPECT scan, 52 of 123 lesions (42%) had a SPECT SUV_max_ greater than 1, of which only 20 of 123 (16%) had an SUV_max_ greater than 3. Of 20 lesions, 15 (75%) with a SPECT SUV_max_ greater than 3 were lymph node (LN) lesions, and 5 of 20 (25%) were bone lesions. Of 52 lesions, 37 LN lesions (71%) and 15 bone lesions (29%) had a SPECT SUV_max_ of greater than 1.

### OARs

**FIGURE 2.**
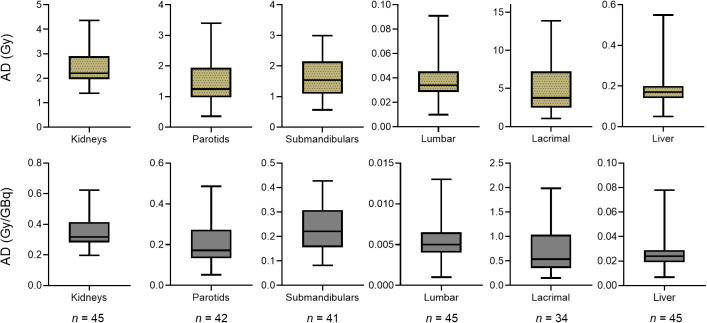
Distribution of AD by organ in grays (top) and grays per gigabecquerel (bottom).

The AD of the kidneys, liver, bone marrow, salivary, and lacrimal glands can be found in Figure 2 and Table 2. In some cases, the submandibular (*n* = 4), parotid (*n* = 3), and lacrimal glands (*n* = 11) were omitted from the AD computation as the glands were out of the field of view in the SPECT/CT or, in a single case, a patient did not have a right submandibular gland. Regarding lumbar vertebrae segmentation as a surrogate for bone marrow exposure, in 3 of 45 (6%) patients, the L4 vertebral body was omitted in the AD computation because of lesion involvement. In all cases, the AD to the kidney and liver was determined.

**TABLE 2.** AD Estimates to OAR

Parameter	Volume (mL)	Mean dose (Gy/GBq)	Mean dose (Gy)
Kidneys*			
Mean ± SD	369.5 ± 74.0	0.35 ± 0.10	2.47 ± 0.72
Median	367.1	0.32	2.23
Range	183.3–555.9	0.20–0.62	1.38–4.36
Liver*			
Mean ± SD	1662.2 ± 356.6	0.03 ± 0.01	0.18 ± 0.09
Median	1605	0.02	0.17
Range	1064.5–2768.8	0.01–0.08	0.05–0.55
Parotid glands^†^			
Mean ± SD	56.5 ± 17.9	0.20 ± 0.10	1.43 ± 0.68
Median	54.3	0.17	1.20
Range	26.2–109.1	0.05–0.49	0.36–3.40
Submandibular glands^‡^			
Mean ± SD	16.1 ± 4.3	0.22 ± 0.11	1.67 ± 1.54
Median	15.8	0.22	1.54
Range	8.7–31.8	0.08–0.43	0.57–2.99
Lacrimal glands^§^			
Mean ± SD	0.7 ± 0.2	0.54 ± 0.52	4.87 ± 3.46
Median	0.7	0.41	3.73
Range	0.3–1.0	0.12–1.98	0.01–13.88
Bone marrow*			
Mean ± SD	75.2 ± 30.1	0.005 ± 0.002	0.038 ± 0.016
Median	75.8	0.005	0.034
Range	9.2–137.6	0.001–0.013	0.010–0.091

* *n* = 45.

† *n* = 42.

‡ *n* = 41.

§ *n* = 34.

### Tumor Lesions

In total, 123 lesions were analyzed: LN (*n* = 82), bone (*n* = 38), and soft tissue (*n* = 3). Patients presented with a median of 2 (range, 1–9) lesions. Median whole-body total tumor volume was 14.5 mL (range, 1.87–145.9 mL) (Table 3). Supplemental Table 3 indicates the difference in the gross tumor volume and PTV.

**TABLE 3.** Distribution of AD by Lesion Type

Parameter	Volume (mL)	Mean dose (Gy/GBq)	Mean dose (Gy)
Whole tumor volume*			
Mean ± SD	20.7 ± 25.3	0.41 ± 0.65	2.84 ± 4.54
Median	14.0	0.16	1.12
Range	1.9–145.9	0.01–3.36	0.05–23.49
LN lesions^†^			
Mean ± SD	5.2 ± 5.2	0.46 ± 0.81	3.19 ± 5.67
Median	3.9	0.15	1.06
Range	1.0–41.1	0.01–5.25	0.09–36.73
Bone lesions^‡^			
Mean ± SD	12.2 ± 10.5	0.19 ± 0.42	1.32 ± 2.96
Median	9.3	0.02	0.15
Range	1.9–47.7	0.01–2.28	0.04–15.94
Soft-tissue lesions^§^			
Mean ± SD	9.3 ± 7.1	0.30 ± 0.38	2.10 ± 3.28
Median	4.8	0.04	0.27
Range	3.8–19.3	0.02–0.84	0.13–5.88

* *n* = 45.

† *n* = 82.

‡ *n* = 38.

§ *n* = 3.

**FIGURE 3.**
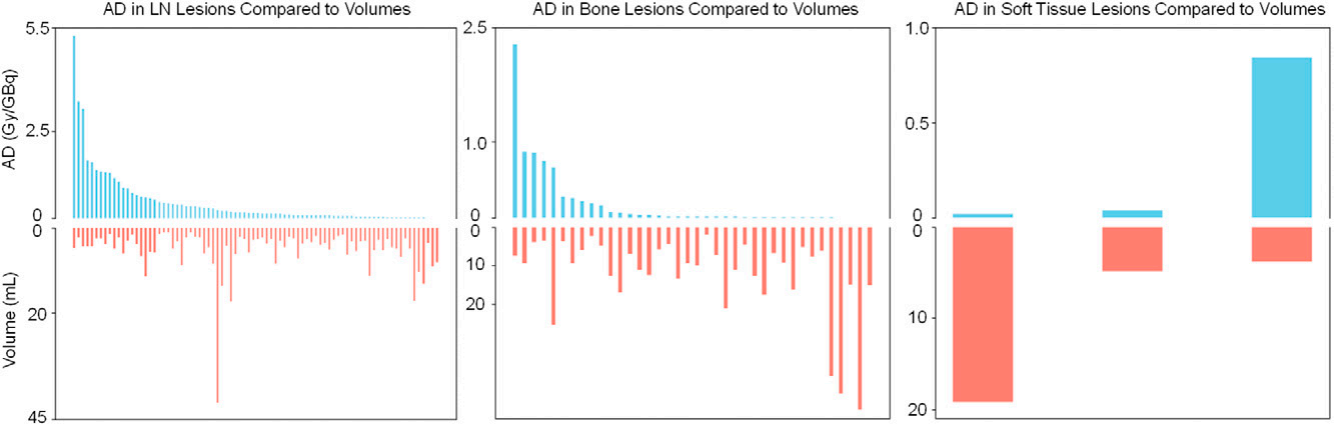
All lesions in study are represented by a bar. No relationship shown between AD (top, blue) and lesion volume (bottom, red). Each panel represents different anatomic location.

The distribution of ADs by lesions is summarized in Table 3. Using cutoffs of more than 1 Gy/GBq and more than 1 Gy for the entire tumor burden, 5 of 45 patients (11%) and 23 or 45 patients (51%), respectively, received an AD above these thresholds. When lesions were evaluated individually, 53 of 123 (43%) had an AD greater than 1 Gy. An AD greater than 1 Gy/GBq was observed in 11 of 82 (13%), 1 of 38 (2%), and 0 of 3 of LN, bone, and soft-tissue lesions, respectively. The AD in 19 of 123 (15%) lesions from 14 different patients were found to be greater than 5 Gy. No dependency between lesion volume and AD was found (*r* = −0.21, *P* = 0.05 for LN; *r* = −0.38, *P* = 0.02 for bone; *r* = −1.0, *P* = 0.33 for soft tissue) (Fig. 3). There was vast heterogeneity in the AD estimates not only on an interpatient level (0.01–36.73 Gy), as shown by the ranges in Table 3, but also on an intrapatient level (range, 0.27–22.14 Gy) across 4 lesions in a representative patient.

### Toxicity and PFS Statistical Analysis

The frequencies of relevant toxicity in the investigational arm compared with the standard of care can be found in Supplemental Table 5. Further investigation of toxicity between the 2 groups can be found in the clinical findings’ publication (14). Any grade anemia (31.1%) and lymphopenia (56.6%) accounted for the most common toxicities in the experimental arm of the study. Neutropenia and thrombocytopenia were not frequent. For the patients who reported xerostomia (*n* = 3) and dry eyes (*n* = 2), AD estimates to the salivary and lacrimal glands were in the bottom third quartile of the respective AD calculation.

Bone marrow–related toxicities and ADs can be found in Table 4. A statistically significant difference (*P* = 0.048) in bone marrow AD was observed between patients who developed grade 1 anemia and those without this development. Although the reported median AD was the same in the adverse event group as in the healthy group, the interquartile range was greater in the former. Of the 14 patients who experienced grade 1 anemia, 3 (21%) had bone lesion involvement and the remainder had only LN disease. Patients who experienced any grade lymphopenia experienced similar bone marrow AD deposition estimates as those who did not (*P* = 0.545). Although patients with any grade neutropenia (*n* = 6) or thrombocytopenia (*n* = 5) received higher doses compared with the groups with neither, limited outcome data precluded statistical confirmation. Patients that experienced any form of bone marrow–related adverse event had a statistically significant higher AD delivered as shown in last row of Table 4 (*P* = 0.0413).

**TABLE 4.** Bone Marrow–Related Toxicity: AD Analysis

Toxicity outcome	Group	Dose (Gy/GBq)	Wilcoxon *P*
Anemia	None	0.005 (0.004–0.006)	0.0484
	Grade 1	0.005 (0.005–0.009)	
Lymphopenia	None	0.005 (0.004–0.006)	0.5450
	Grade 1	0.005 (0.004–0.006)	
Neutropenia	None	0.005 (0.004–0.006)	0.2867
	Grade 1+	0.006 (0.005–0.009)	
Thrombocytopenia	None	0.005 (0.004–0.006)	0.9070
	Grade 1+	0.006 (0.004–0.008)	
At least one toxicity	None	0.004 (0.003–0.005)	0.0413
	Grade 1+	0.005 (0.004–0.007)	

Continous data are median with interquartile range in parentheses.

Evaluation of whole tumor burden AD and progression is shown in Supplemental Figure 3 and Supplemental Table 6, respectively.

## DISCUSSION

In the LUNAR trial, the addition of [^177^Lu]Lu-PSMA-I&T to SBRT in patients with omHSPC significantly improved PSMA PET/CT–based PFS. In this dosimetry analysis of 45 patients, we report the ADs from [^177^Lu]Lu-PSMA-I&T to OARs remained within expected tolerable limits and were comparable to those in published studies of [^177^Lu]Lu-PSMA-I&T (19). Specifically regarding omHSPC, only a few studies exist, and these evaluate [^177^Lu]Lu-PSMA-617. Although AD estimates to OARs were similar, tumor ADs varied from those in this work (Supplemental Table 7) (8,13,20,21). Although lesion ADs were determined to be low, 16% of patients had a 30% or greater reduction in at least one target lesion, prompting a reduction in PTV and dose adjustment for SBRT treatment. Two patients (4%) had a complete resolution of all disease radiographically. One (2%) patient had progressive disease on the post–[^177^Lu]Lu-PSMA-I&T interval PSMA PET/CT scan, manifesting as multiple new lesions; this patient did not receive SBRT (14).

In 23 of 45 patients (51%), the total tumor burden AD was greater than 1 Gy, and when lesions were evaluated individually, 53 of 123 lesions (43%) had an AD of greater than 1 Gy. The AD in 19 of 123 lesions (15%) from 14 different patients was found to be greater than 5 Gy.

To date, there are very few dosimetry publications (19) for patients with omHSPC (8,13,20,21), let alone for those with low-volume disease (<145 mL total) and multiple-time-point SPECT/CT. This is the first study that evaluates the AD to men with omHSPC included in a registered clinical trial within the United States.

Safety and tolerability are currently being investigated as RPT regimes evolve, including in combination with SBRT. The ADs to OAR remained below accepted limits (22). Five patients reported xerostomia (*n* = 3) or dry eyes (*n* = 2), yet calculated AD estimates to the salivary and lacrimal glands, respectively, were in the bottom third quartile of the cohort. Significant partial volume error, patient movement during imaging, and subjectivity in patient reporting may contribute to the uncertainty in the assessment. There were 14 anemia-related events in the [^177^Lu]Lu-PSMA-I&T cohort in this study. Despite this small number of patients, we found a statistically significant difference between the calculated bone marrow AD for those patients who experienced anemia versus the group who did not (Table 4). However, this was not the case for lymphopenia, which may be noteworthy given the number of productive rearrangements in the T-cell receptor at 90 d has been found to be prognostic in men receiving metastasis-directed therapy (14,23). It should be noted that the bone marrow AD calculation was image-based and used a surrogate segmentation of the lumbar vertebrae.

In our analysis, the tumor sink effect was not identified. That is, because of the small overall tumor burden, one may hypothesize that the organs would receive a greater AD, which was not the case in this analysis. However, accurate reporting of AD to the lacrimal and salivary glands remains difficult because of activity spillover in the small volumes and patient head movement during SPECT acquisition.

A combined dosimetry study of [^177^Lu]Lu-PSMA-I&T plus SBRT is under way to assess cumulative ADs to lesions and potential additive effects to healthy tissues. Evaluation of combined dosimetry from different irradiation mechanisms is challenged by the accuracy of conversion from AD to biologic effective dose (24–26) and lacks existing clinical data for validation.

### Limitations

The tumor volume that was used in the AD computation was produced from the SBRT PTV treatment plan, meaning that both cycles of [^177^Lu]Lu-PSMA-I&T had been administered already. There is possibility of disease shrinkage or progression in that time, as demonstrated in 7 of 45 patients (16%) in our study that had a target volume reduction for SBRT based on the [^177^Lu]Lu-PSMA-I&T treatment.

The use of a monoexponential curve fit, which occasionally showed poor fitting in lesions (36/123 [29%], *R*^2^ < 0.5), arose because the first SPECT time point did not always represent maximum uptake within the lesion, underscoring that reported ADs are approximations requiring nuanced interpretation and evaluation.

A patient population characterized by small-volume disease challenges the spatial resolution limits of PET/CT and SPECT/CT, potentially affecting SUV measurements and AD estimates. Because of the limited spatial resolution of SPECT, partial-volume effects impair accurate activity quantification in small structures (e.g., tumors, salivary and lacrimal glands) unless corrected (27). Although we did account for partial-volume effects using spheric phantom-based corrections, these may not accurately reflect the uptake of all lesions. Low uptake, or low-contrast lesions, may present a challenge for accurate activity quantification. Although this lack of detectable signal limits the feasibility of accurate dosimetry, this should not imply that no therapeutic radiation was delivered, especially given the limitations of SPECT imaging. Indeed, the outcomes from the clinical findings’ publication (14) suggest a therapeutic effect from the addition of [^177^Lu]Lu-PSMA-I&T acting on small-volume, and occult, undetectable disease.

## CONCLUSION

This study provides the first AD analysis of [^177^Lu]Lu-PSMA-I&T in patients with low-volume (20.7 ± 25.3 mL) omHSPC. The AD to OAR remained within expected tolerable limits and showed no signal of a tumor sink effect. The ADs to tumors were vastly heterogeneous within and among the patient cohort. The limited SPECT spatial resolution leads to partial-volume effects that can underestimate the AD in small lesions of patietns with omHSPC. Importantly, 2 neoadjuvant cycles of [^177^Lu]Lu-PSMA-I&T before SBRT still prolonged PFS, consistent with effective [^177^Lu]Lu-PSMA-I&T treatment of occult disease beyond SPECT imaging detectability.

## DISCLOSURE

Lena Unterrainer reports fees from Novartis (speaker), Telix (consultant), and Astellas Pharma Inc. (speaker) outside of the submitted work. Minsong Cao received personal fees from Varian Inc. and Medical Dosimetry Certification Board outside of the submitted work. Johannes Czernin is a founder of SOFIE Biosciences and holds equity in the company and in intellectual property invented by him, patented by the University of California, and licensed to SOFIE Biosciences. He is a founder and board member of Trethera Therapeutics and holds equity in the company and in intellectual property invented by him, patented by the University of California, and licensed to Triangle. He serves on the medical advisory board of Actinium Pharmaceuticals and on the scientific advisory boards of POINT Biopharma, RayzeBio, and Aktis Oncology. Jeremie Calais reports grant support to his institution from Novartis, Lantheus, Fusion Pharmaceuticals, Telix Pharmaceuticals, and POINT Biopharma. He also reports consulting activities (advisory boards, speaker, blinded reader) for Advanced Accelerator Applications, Advancell, Amgen, Arsenal Bio, Astellas, Bayer, Blue Earth Diagnostics, Cardinal Health, Curium Pharma, Coretag, DS Pharma, Fibrogen, GE HealthCare, Isoray, IBA RadioPharma, Janssen Pharmaceuticals, Keosys, Lightpoint Medical, Lantheus, Monrol, Mariana Oncology, Novartis, Nucleus Radiopharma, Nucs AI, OranoMed, Pfizer, POINT Biopharma, Progenics, Radiomedix, Radiopharm Theranostics, Sanofi, Siemens-Varian, SOFIE, and Telix Pharmaceuticals outside of the submitted work. Amar Kishan reports consulting activities from Janssen, Boston Scientific, and Lantheus Medical Imaging and research funding from POINT Biopharma. No other potential conflict of interest relevant to this article was reported.

## Notes

KEY POINTS **QUESTION:** What effect does [^177^Lu]Lu-PSMA-I&T have in patients with early disease stage (omHSPC) in terms of AD to tumors and OAR? **PERTINENT FINDINGS:** The AD to OARs remained within tolerable limits, comparable to past publications, and had no sign of an increase because of the low overall tumor burden (lack of tumor sink effect). Although the ADs to tumors were vastly heterogeneous on both an intra- and interpatient level, the PFS after SBRT in this cohort was increased with the addition of [^177^Lu]Lu-PSMA-I&T. Dosimetry of low-volume, low-uptake lesions is challenged by spatial resolution and partial-volume effects of SPECT imaging, likely contributing to the low overall estimated tumor ADs and not reflective of the biological effective dose deposition of the therapy. **IMPLICATIONS FOR PATIENT CARE:** Contrary to the tumor sink effect, the low overall tumor burden did not increase the AD to OARs in this cohort of patients. Although individual tumor doses were vastly heterogeneous, the population of patients with omHSPC in the LUNAR clinical trial found beneficial PFS outcomes with 2 cycles of [^177^Lu]Lu-PSMA-I&T added prior to SBRT.
